# Breastfeeding Difficulties and Risk for Early Breastfeeding Cessation

**DOI:** 10.3390/nu11102266

**Published:** 2019-09-20

**Authors:** Maria Lorella Gianni, Maria Enrica Bettinelli, Priscilla Manfra, Gabriele Sorrentino, Elena Bezze, Laura Plevani, Giacomo Cavallaro, Genny Raffaeli, Beatrice Letizia Crippa, Lorenzo Colombo, Daniela Morniroli, Nadia Liotto, Paola Roggero, Eduardo Villamor, Paola Marchisio, Fabio Mosca

**Affiliations:** 1 Fondazione IRCCS Ca’ Granda Ospedale Maggiore Policlinico, NICU, via Commenda 12, 20122 Milan, Italy; priscilla.manfra@gmail.com (P.M.); gabriele.sorrentino@mangiagalli.it (G.S.); elena.bezze@policlinico.mi.it (E.B.); laura.plevani@mangiagalli.it (L.P.); giacomo.cavallaro@mangiagalli.it (G.C.); beatriceletizia.crippa@gmail.com (B.L.C.); lorenzo.colombo@policlinico.mi.it (L.C.); daniela.morniroli@gmail.com (D.M.); nadia.liotto@mangiagalli.it (N.L.); paola.roggero@unimi.it (P.R.); fabio.mosca@mangiagalli.it (F.M.); 2 Department of Clinical Sciences and Community Health, University of Milan, Via San Barnaba 8, 20122 Milan, Italy; maria.bettinelli@unimi.it; 3 Department of Pediatrics, Maastricht University Medical Center (MUMC+), School for Oncology and Developmental Biology (GROW), 6202 AZ Maastricht, The Netherlands; e.villamor@mumc.nl; 4 Fondazione IRCCS Ca’ Granda Ospedale Maggiore Policlinico, 20122 Milan, Italy; paola.marchisio@unimi.it; 5 Department of Pathophysiology and Transplantation, University of Milan, 20122 Milan, Italy

**Keywords:** breastfeeding difficulties, early breastfeeding cessation, term infants, breastfeeding support

## Abstract

Although breast milk is the normative feeding for infants, breastfeeding rates are lower than recommended. We investigated breastfeeding difficulties experienced by mothers in the first months after delivery and their association with early breastfeeding discontinuation. We conducted a prospective observational study. Mothers breastfeeding singleton healthy term newborns at hospital discharge were enrolled and, at three months post-delivery, were administered a questionnaire on their breastfeeding experience. Association among neonatal/maternal characteristics, breastfeeding difficulties and support after hospital discharge, and type of feeding at three months was assessed using multivariate binary logistic regression analysis. We enrolled 792 mothers, 552 completed the study. Around 70.3% of mothers experienced breastfeeding difficulties, reporting cracked nipples, perception of insufficient amount of milk, pain, and fatigue. Difficulties occurred mostly within the first month. Half of mothers with breastfeeding issues felt well-supported by health professionals. Maternal perception of not having a sufficient amount of milk, infant’s failure to thrive, mastitis, and the return to work were associated with a higher risk of non-exclusive breastfeeding at three months whereas vaginal delivery and breastfeeding support after hospital discharge were associated with a decreased risk. These results underline the importance of continued, tailored professional breastfeeding support.

## 1. Introduction

Breastfeeding is associated with improvement of infants’ survival and significant health benefits both for infants and mothers in a dose-response manner [1,2,3]. Consequently, promotion and support of breastfeeding initiation, duration, and exclusivity is a public health issue. However, the worldwide rates of breastfeeding are lower than international recommendations, especially in high-income countries [4]. Therefore, there is a need for increasing the health care professionals’ awareness of the intrinsic factors associated with early breastfeeding cessation and for gaining further insight into the related modifiable risk factors [5]. Several determinants of breastfeeding have been described within a complex framework, including structural settings and individual factors that are involved at multiple levels [6]. Among the individual factors, the experience of breastfeeding difficulties greatly contributes to early breastfeeding cessation and causes mothers to be less likely to breastfeed a future child [7]. However, “breastfeeding difficulties” includes a wide range of different biological, psychological, and social factors [8]. Unpacking this issue to gain further insight into the modifiable barriers mothers experience during breastfeeding may help health professionals in overcoming them and in refining community support [5].

The aim of the present study was to investigate the breastfeeding difficulties experienced by mothers of healthy, singleton term-born infants in the first months after delivery and their association with early breastfeeding discontinuation.

## 2. Materials and Methods

We conducted a prospective, observational study in the nursery of Fondazione IRCCS Ca’ Granda Ospedale Maggiore Policlinico in Milan, Lombardy, Italy. The hospital is a Level III center for neonatal care that covers around 6000 deliveries per year, admitting pregnant women prevalently resident in Lombardy but also those resident in other Italian regions.

All subjects gave their informed consent for inclusion before they participated in the study. The study was conducted in accordance with the Declaration of Helsinki, and the protocol was approved by the Ethics Committee of Milano (Comitato Etico Milano Area 2, n. 0120, atti n. 1580/2018).

Mothers with a low risk for early breastfeeding cessation, that is having delivered singleton, healthy, term (gestational age ≥37 weeks) newborns with the birthweight ≥10th percentile for gestational age, according to the Bertino’s neonatal growth chart [9], and breastfeeding were enrolled at hospital discharge, which occurred within the completion of the first 72 h after delivery. Exclusion criteria included exclusive formula feeding, multiple pregnancy, non-Italian speaking mothers due to fact that the language barrier could have interfered with the accuracy of the answers, and mothers whose newborns were admitted to Neonatal Intensive Care Unit and/or were affected by any condition that could interfere with breastfeeding, such as congenital diseases, chromosomal abnormalities, lung disease, brain disease, metabolic disease, cardiac disease, or gastrointestinal diseases. Breastfeeding was promoted and supported in all mother-infant pairs throughout the hospital stay, following the Ten Steps to Successful Breastfeeding [10]. Socio-demographic maternal variables (age, marital status, education, mode of delivery, parity), basic infants’ characteristics (gestational age, birth weight, length, head circumference, Apgar score), and the infants’ mode of feeding at hospital discharge were collected. At discharge, mothers were instructed to record in a diary their infant’s mode of feeding at seven days, one month, and three months after delivery. The mode of feeding was categorized according to the World Health Organization definition [11] as exclusive breastfeeding (infants are fed only breast milk and no other food or drink; not even water; oral rehydration solutions, drops and syrups such as vitamins, minerals and medicines are permitted); predominant breastfeeding (breast milk is the infant’s predominant source of nourishment but liquids such as water and water-based drinks are permitted); complementary feeding (infants are mainly breastfed but also consume formula milk and other liquid or non-dairy foods); and exclusive formula feeding.

At three months post-delivery, mothers were contacted by phone in order to collect the recorded infant feeding data, and were reminded to access and complete the online questionnaire investigating their breastfeeding experience following hospital discharge within the subsequent 48 h. Specifically, mothers were asked whether they had encountered any difficulty with regard to breastfeeding. If the mothers answered yes, they had to report which difficulties they had encountered during their breastfeeding experience, when the encountered difficulties had arisen (discharge–1st month after delivery, 1st month–2nd month after delivery, 2nd month–3rd month after delivery) and how they had been solved. Mothers were also required to rate the breastfeeding support they had received by health care professionals after hospital discharge (excellent, very good, satisfactory, poor, very poor, or unacceptable).

### Statistical Analysis

Data are presented as mean (SD) or number of observations (%). For analysis, maternal age was divided into two categories based on the median value; maternal educational age was categorized as ≤13 years or >13 years, while breastfeeding support after discharge was considered positive if mothers rated it either as excellent, very good, or satisfactory and negative if the mothers rated it either as poor, very poor or unacceptable. Mode of feeding was categorized as exclusive breastfeeding vs. non-exclusive breastfeeding. The latter category included complementary feeding and exclusive formula feeding. Association between socio-demographic characteristics, the mode of delivery, parity, the occurrence of breastfeeding difficulties at any time point of the study, having been supported after hospital discharge and the mode of infant’s feeding at three months (reference group: non-exclusive breastfeeding) were first assessed using univariate binary logistic regression analysis. A multivariate binary logistic regression analysis was then conducted in order to identify which breastfeeding difficulties arisen through the study period were independently associated with the type of feeding at three months. When adjusting the model, we included the items that showed a significant association with type of feeding at univariate analysis. Statistical significance was set at the α = 0.05 level. The statistical analyses were performed using SPSS (version 12, SPSS, Inc., Chicago, IL, USA).

## 3. Results

Of the 1843 mothers who delivered during the study period, 868 were eligible for the study. A total of 76 mothers refused to participate and 792 mother-infant pairs were enrolled. Among these, 552 (70%) completed the study and the online questionnaire whereas the remaining 240 mothers did not complete either the study or the online questionnaire since it was not possible to reach them by telephone after hospital discharge.

Basic characteristics of mother-infant pairs which completed the study are summarized in Table 1. Mother-infant pairs that have not completed the study did not significantly differ from the ones completing the study.

The mode of feeding at each time point of the study is reported in Table 2. At enrollment, 95% of the mothers practiced exclusive breastfeeding, whereas 5% of the mothers practiced complementary feeding. At one and three months, exclusive breastfeeding rates declined to 73% and 68%, respectively, whereas complementary feeding rates were 20% and 15%, respectively. Percentage of infants receiving exclusive formula feeding was 7% at one month, increasing up to 17% at three months.

A total of 388 (70.3%) mothers experienced difficulties during breastfeeding. The difficulties most frequently reported by the mothers were cracked nipples, the perception of insufficient amount of milk, pain, and fatigue (Table 3).

Most of the mothers (63%) reported the occurrence of difficulties within the first month after delivery whereas, in the second and third month after delivery, difficulties were experienced only by 9% and 10% of the enrolled mothers, respectively. A total of 189 (48.7%) mothers among those that have encountered difficulties in breastfeeding reported they were successfully supported by health professionals, whereas 78 (20.1%) mothers solved the difficulties by themselves and 45 (11.6%) mothers with the support of friends or relatives. Difficulties were not solved in 19.6% of cases; however, 7% of these latter mothers kept on breastfeeding.

After hospital discharge, the breastfeeding support received by health professionals was rated as either excellent, very good, or satisfactory in most cases (86.1%) whereas only in the 13.9% of cases the breastfeeding support was reported as either poor, very poor, or unacceptable. The mothers who rated the breastfeeding support after hospital discharge as negative were at higher risk of non-exclusive breastfeeding at three months than the mothers that rated the support after hospital discharge as positive (OR = 1.367, 95%CI 1.09–1.70, *p* = 0.005).

Univariate analysis showed that the absence of breastfeeding difficulties and having been supported in case of difficulties were significantly associated with a lower risk of non-exclusive breastfeeding at three months (OR = 0.051; 95% CI 0.022; 0.117, *p* < 0.0001; OR = 0.39; 95% CI 0.202–0.756, *p* = 0.005, respectively). When taking into account the type of breastfeeding difficulties, the perception of not having enough milk, pain perception, infant’s failure to thrive, the perception of milk’s limited nutritional value, the occurrence of mastitis, and the return to work were associated with a higher risk of non-exclusive breastfeeding at three months (Table 4). Primiparity and an incorrect latching tended to be associated with a higher risk of non-exclusive breastfeeding at three months whereas vaginal delivery resulted in being associated with a lower risk (Table 4). No significant association was found between maternal education level and age, breast engorgement, cracked nipples, fatigue, prescription drugs, and the infant’s mode of feeding (Table 4).

Multivariate binary logistic regression showed that the maternal perception of not having a sufficient amount of milk, infant’s failure to thrive, mastitis, and the return to work were associated with a higher risk of non-exclusive breastfeeding at three months whereas vaginal delivery and breastfeeding support after hospital discharge were associated with a decreased risk (Table 5).

## 4. Discussion

Increasing awareness of the modifiable barriers experienced by mothers during breastfeeding may help health professionals in the detection of mothers at risk for early cessation of breastfeeding and the implementation of targeted breastfeeding support [12,13].

Our findings contribute to the understanding of the specific breastfeeding difficulties experienced by mothers with a low risk for early breastfeeding cessation, which appear to be related to several major areas, including lactational, nutritional, psychosocial, lifestyle, and medical factors, towards which breastfeeding promotion and support at the community level should be directed. Indeed, although in our study, the mother–infant dyads were enrolled in only one hospital, the present results reflect the primary care provided by the national “family pediatrics” network at the community level since, according to the Italian Public Health Care System, all patients aged 0–16 years must have an identified primary care provider among those available in the different regional health districts [14].

The perception of not having enough milk, the infant’s failure to thrive, and mastitis are well-known factors acting negatively on breastfeeding [15,16,17,18,19], according to our results. Moreover, in this study, the return to work was associated with early exclusive breastfeeding failure. As previously described, balancing work and exclusive breastfeeding is challenging and requires a strong support in the short and long term [20,21]. In this scenario, employers could play a critical role in providing encouragement for working mothers to continue breastfeeding after returning to work and workplaces should establish dedicated breastfeeding rooms [22,23,24,25,26].

The perception of milk’s limited nutritional value and pain during lactation was associated with a higher risk of exclusive breastfeeding discontinuation only in univariate analysis. It can be speculated that these factors might be closely related to the perception of reduced milk supply and often mentioned together. Incorrect latching showed a tendency even though it did not reach statistical significance, possibly reflecting the provision of adequate education and support both during the hospital stay and after hospital discharge with regard to the improvement of mothers’ breastfeeding technique.

The findings of the present study are consistent with previous studies in the literature. Poor breastfeeding technique has been reported among the individual factors associated with unsuccessful breastfeeding [6,15,24,27], indicating that adequate breastfeeding support, including evaluation of latching, position, and feeding at the breast, could prevent nipple cracks and thus mastitis. Accordingly, the impact on breastfeeding cessation of acute pain, fever, and other typical mastitis symptoms presented by 8–10% of breastfeeding mothers has been broadly described in literature [28,29,30]. Mosca et al. [31] found that lactational and nutritional factors were the most cited by mothers as determinants for breastfeeding discontinuation, particularly during the first three months after delivery. Remarkably, the authors reported that the evaluation by a health care professional was rated as important only in 29% to 51% of cases whereas the maternal perception of inadequate milk or insufficient milk supply was cited as important by 40% up to 99% of mothers through the six months’ study duration.

The present findings highlight the importance of educating mothers on the criteria that have to be taken into account when considering the adequateness of breast milk supply. Moreover, in this study, our results confirm the association between infant’s failure to thrive and discontinuation of exclusive breastfeeding at three months. Accordingly, it has also been described how infant’s failure to thrive, objectively evaluated by a healthcare professional, was one of the reasons of exclusive breastfeeding discontinuation, reported throughout the first 6 months of lactation [31]. Interestingly, a study by Flaherman et al. [32] has reported how early and limited administration of small quantities of formula milk during hospital stay could improve breastfeeding rates at three months. The authors speculated that limiting infants’ weight loss during the first days of life may reduce maternal milk supply concern, which has been associated with breastfeeding discontinuation. It is then crucial to enhance maternal confidence in her own abilities, enabling mothers to get further insight into the lactation process and the peculiar characteristics of infant growth that often take place in spurts [16]. Within this context, it has to be underlined that a previous negative breastfeeding experience and difficulty negatively affect the likelihood of subsequent breastfeeding success, leading to a potential fear of breastfeeding secondary to prior breastfeeding trauma [7].

In agreement with previous data [15,27,31], in the present study, mothers reported psychosocial factors, in terms of pain and fatigue as breastfeeding difficulties in a relatively high number of cases. The occurrence of physical difficulty during breastfeeding has been associated with a greater risk for developing depressive symptoms in the postnatal period. Hence, it is crucial to provide mothers with early adequate breastfeeding support, including emotional [8].

Accordingly, antenatal and postnatal support including mothers’ counseling and education positively affects breastfeeding success [6,12]. Consistently, in the present study, the availability of adequate support at the community level was associated with exclusive breastfeeding at three months post-delivery. Moreover, our results confirm that the mode of delivery modulates breastfeeding success [33], although it must be considered that caesarean section does not seem to negatively impact breastfeeding outcomes at six months, once adequate breastfeeding support is provided [34].

On the contrary, no mention about lifestyle factors, previously reported by other authors, regarding body image, such as wish to lose weight or dislike of breast appearance and breastfeeding convenience [8,15], have been reported, suggesting a positive breastfeeding attitude within the enrolled mothers.

Remarkably, most of the reported breastfeeding difficulties occurred within the first month after delivery, highlighting the importance of offering continuity of care after hospital discharge as underlined in the third guiding principle of the Ten Steps to Successful Breastfeeding [10]. Moreover, the largest decrease in exclusive breastfeeding in the present study was registered between enrollment and seven days after birth.

Literature shows how global breastfeeding rates are far below the international targets, particularly for high-income countries [4], although Italy has one of the highest rates of early initiation of breastfeeding. Moreover, according to the Italian National Statistics Institute [35], in Italy, 48.7% of infants are being exclusively breastfed in the first month, with a drop to 43.9% within the first three months. A survey conducted in 2012 in Lombardy [36] reported a progressive reduction of exclusive breastfeeding rates from 67.3% at hospital discharge to 47.3% and 27% within 120 and 180 days, respectively. Our rates are higher and reflect a particular local context of a high-income country where the breastfeeding benefits are well known and mothers are also supported at the community level. It must be acknowledged that this study focused on mothers with a low risk for breastfeeding cessation and did not include non-Italian speaking mothers due to the potential language barrier that could have interfered with the accuracy of the results, even though they could actually represent a subgroup particularly in need of breastfeeding support structures.

The strength of the present study is that it enrolled a relatively large sample of breastfeeding mother-infant pairs even though the duration of follow up was relatively limited and the dropout rate was 30%, thus partially limiting the generalizability of the present findings. However, it has to be taken into account that, with regard to cohort studies, although the maximum follow-up rate possible should be achieved, dropout rates ranging from 20% up to 50% have been suggested as acceptable [37].

## 5. Conclusions

Our findings provide further insight into breastfeeding difficulties experienced by mothers through the first three months after delivery in a high-income country with a positive breastfeeding culture and attitude. We underline the importance of providing continued tailored professional support in the community in the attempt to overcome maternal breastfeeding difficulties after discharge from the hospital.

## Figures and Tables

**Table 1:** Basic characteristics of the mother-infant pairs that completed (*n* = 552) and that not completed (240) the study.

**Mothers that Completed the Study (*n* = 552)**		**Mothers Who did not Complete the Study (*n* = 240)**
Maternal age, years (mean ± SD)	35.5 ± 4.6	34.9 ± 4.6
Marital status, *n* (%)		
Married or cohabitant	540 (98)	237 (99)
Single or divorced	12 (2)	3 (1)
Maternal education level, *n* (%)		
≤13 years	150 (27)	75 (31)
>13 years	402 (73)	165 (69)
Vaginal delivery, *n* (%)	369 (66.8)	157 (65.4)
Primiparous, *n* (%)	290 (52.5)	138 (57.5)
**Infants Born to Mothers that had Completed the Study (*n* = 552)**		**Infants Born to Mothers Who did not Complete the Study (*n* = 240)**
Gestational age, weeks (mean ± SD)	39.2 ± 1.0	39.3 ± 0.9
Birth weight, g (mean ± SD)	3368 ± 350	3390 ± 332
Length, cm (mean ± SD)	50.1 ± 1.6	50.3 ± 1.5
Head circumference, cm (mean ± SD)	34.5 ± 1.4	34.3 ± 1.3

**Table 2:** Mode of feeding at each time point of the study.

	Enrollment	Seven Days	One Month	Three Months
Exclusive breastfeeding	524 (95%)	447 (81%)	402 (73%)	375 (68%)
Predominant breastfeeding	0%	5 (1%)	5 (1%)	5 (1%)
Complementary feeding	28 (5%)	99 (18%)	105 (19%)	77 (14%)
Exclusive formula feeding	0%	0%	39 (7%)	94 (17%)

**Table 3:** Breastfeeding difficulties arisen at any time point of the study according to mothers’ experience.

Breastfeeding Difficulties	*N* (%)
Cracked nipples	159 (41.0)
Perception of an insufficient amount of milk	139 (35.8)
Pain not associated with cracked nipples	121 (31.2)
Fatigue	117 (30.2)
Breast engorgement	102 (26.3)
Infant’s failure to thrive	79 (20.4)
Incorrect latching	74 (19.1)
Perception of own’s milk limited nutritional value	68 (17.5)
Mastitis	27 (7.0)
Return to work	17 (4.4)
Prescription drugs	8 (2.1)

**Table 4:** Association among maternal age and education, the mode of delivery, parity, and types of breastfeeding difficulties and the mode of infant’s feeding at three months (univariate binary logistic regression analysis).

Reference Group: Non-Exclusive Breastfeeding			
	OR	95%; CI	*p*
Maternal age (≤35 vs. >35 years)	1.02	0.711; 1.465	0.913
Maternal education (≤13 vs. >13 years)	0.67	0.237; 1.93	0.465
Mode of delivery (spontaneous vs. caesarean delivery)	0.60	0.415; 0.881	0.009
Parity (primiparous vs. multiparous)	1.42	0.988; 2.051	0.058
Cracked nipples (yes vs. no)	1.38	0.933; 2.042	0.107
Perception of not having enough milk (yes vs. no)	9.23	5.961; 14.301	<0.0001
Pain not associated with cracked nipples (yes vs. no)	1.62	1.066; 2.487	0.024
Fatigue (yes vs. no)	1.22	0.790; 1.903	0.363
Breast engorgement (yes vs. no)	0.87	0.545; 1,412	0.590
Infant’s failure to thrive (yes vs. no)	5.136	3.094; 8.525	<0.0001
Incorrect latching (yes vs. no)	1.58	0.949; 2.635	0.078
Perception of milk’s limited nutritional value (yes vs. no)	3.44	2.015; 5.898	<0.0001
Mastitis (yes vs. no)	2.49	1.144; 5.420	0.022
Return to work (yes vs. no)	7.65	2.457; 23.830	<0.0001
Prescription drugs (yes vs. no)	2.29	0.266; 19.761	0.452

**Table 5:** Association among the mode of delivery, having been supported after discharge, the types of breastfeeding difficulties and the mode of infant’s feeding at three months (multivariate binary logistic regression analysis).

	Reference Group: Non-Exclusive Breastfeeding			
	B	OR	95%; CI	*p*
Mode of delivery (spontaneous vs. caesarean delivery)	−0.57	0.56	0.329; 0.961	0.035
Having been supported after hospital discharge (yes vs. no)	−1.28	0.27	0.130; 0.594	0.001
Perception of not having enough milk (yes vs. no)	1.96	7.15	4.096; 12.499	<0.0001
Pain not associated with cracked nipples (yes vs. no)	0.25	1.29	0.737; 2.265	0.37
Infant’s failure to thrive (yes vs. no)	1.00	2.73	1.441; 5.180	0.002
Perception of milk’s limited nutritional value (yes vs. no)	0.59	1.81	0.912; 3.607	0.089
Mastitis (yes vs. no)	1.07	2.92	1.166; 7.314	0.022
Return to work (yes vs. no)	1.63	5.136	1.046; 25.204	0.044

## Abbreviations

SD	Standard Deviation,
OR	Odd Ratio,
CI	Confidence Interval
